# Impact of Immunosuppressants and Vaccination on COVID-19 Outcomes in Autoimmune Patients and Solid Organ Transplant Recipients: A Nationwide Propensity Score-Matched Study

**DOI:** 10.3390/vaccines12101190

**Published:** 2024-10-18

**Authors:** Mindong Sung, Young-Sam Kim, Changjin Cho, Yongeun Son, Dong-Wook Kim, Su-Hwan Lee

**Affiliations:** 1 Division of Pulmonology and Critical Care Medicine, Department of Internal Medicine, Yonsei University College of Medicine, Seoul 03722, Republic of Korea; mdsung@yuhs.ac.kr (M.S.); ysamkim@yuhs.ac (Y.-S.K.); 2 Department of Information Statistics, Gyeongsang National University, Jinju 52828, Republic of Korea; cxz7933@naver.com (C.C.); sye515@gnu.ac.kr (Y.S.); 3 Department of Information and Statistics, Research Institute of Natural Science, Gyeongsang National University, 501 Jinju-daero, Jinju 52858, Republic of Korea; 4 Department of Bio & Medical Bigdata (BK21 Plus), Gyeongsang National University, Jinju 52828, Republic of Korea

**Keywords:** COVID-19, immunocompromised, vaccination, autoimmune disease, solid organ transplantation

## Abstract

Purpose: This study investigates the impact of varying degrees of immunosuppression on the clinical outcomes of immunocompromised individuals, particularly those with autoimmune diseases or post-solid organ transplant statuses, in the context of COVID-19. By focusing on these highly vulnerable populations, the study underscores the significant health inequalities faced by immunocompromised patients, who experience disproportionately worse outcomes in comparison to the general population. Methods: A retrospective cohort analysis of the K-COV-N dataset was conducted, comparing the effects of immunosuppression in autoimmune and transplant groups with matched control groups. Propensity score matching was employed to minimize inequalities in baseline characteristics, ensuring a more equitable comparison between immunocompromised and non-immunocompromised individuals. Outcomes included COVID-19-related in-hospital mortality, 28-day mortality, ICU admissions, and the need for respiratory support among 323,890 adults in the Republic of Korea. Patients with cancer or other immunosuppressive conditions, such as HIV, were excluded. Subgroup analyses assessed the influence of specific immunosuppressive medications and vaccination extent. Results: Significantly elevated in-hospital mortality was found for patients with autoimmune diseases (adjusted Odds Ratio [aOR] 2.749) and transplant recipients (aOR 7.567), with similar patterns in other outcomes. High-dose steroid use and a greater number of immunosuppressant medications markedly increased the risk of poor outcomes. Vaccination emerged as a protective factor, with a single dose substantially improving outcomes for autoimmune patients and at least two doses necessary for transplant recipients. Conclusions: Immunocompromised patients, particularly those with autoimmune diseases and transplant recipients, are highly vulnerable to severe COVID-19 outcomes. High-dose steroid use and multiple immunosuppressants further increase risks. Vaccination significantly improves outcomes, with at least one dose benefiting autoimmune patients and two doses necessary for transplant recipients. Personalized vaccination schedules based on immunosuppression levels are essential to mitigate healthcare inequalities and improve outcomes, particularly in underserved populations, informing both clinical and public health strategies.

## 1. Introduction

The Coronavirus Disease (COVID)-19 pandemic, instigated by the discovery of Severe Acute Respiratory Syndrome Coronavirus-2 (SARS-CoV-2) in late 2019, poses an unprecedented challenge to global health [1,2,3] Immunocompromised patients are among the most vulnerable to this virus. Their heightened susceptibility is attributed to their inherently weakened immune system, which is often exacerbated by immunosuppressive medications that are administered to them [4,5]. Specifically, transplant recipients typically require higher doses of these medications than patients with other autoimmune diseases, which needs to be considered when evaluating their response to treatments and preventive measures such as vaccinations.

The clinical impact of immunosuppression on COVID-19 outcomes has been the focus of multiple studies [5,6,7,8,9], particularly in cohorts of patients with autoimmune diseases [10,11] and solid organ transplants [4,12,13,14,15,16,17]. Research consistently demonstrates that immunosuppressed individuals face higher risks of severe disease, hospitalization, and mortality due to their impaired immune responses. However, previous studies have notable limitations. Many have not provided detailed analyses of how specific immunosuppressive medications interact with COVID-19 outcomes or vaccination responses, and some have been constrained by small sample sizes or heterogeneous populations, limiting the generalizability of their findings. Despite these limitations, prior research has laid a critical foundation in understanding the heightened risks faced by immunocompromised patients.

As the severity of the pandemic became evident, global efforts quickly prioritized the development of vaccines [18,19,20,21]. Research indicates that immunocompromised individuals may exhibit a diminished immune response to vaccines, leaving them more vulnerable to severe COVID-19 despite vaccination [22,23,24,25]. Reflecting this, the institutions such as the US FDA and CDC have updated their guidelines, recommending additional vaccine doses for individuals with weakened immune systems [26,27]. Current guidelines strongly promote vaccination due to its proven role in preventing hospitalizations and reducing the severity of illness [28].However, uncertainty persists regarding the outcomes of COVID-19 in immunocompromised patients, particularly concerning their response to vaccination [9,12,13,29]. The variability in immune status within this population and the incomplete understanding of vaccine efficacy contribute to this uncertainty [30].

This study investigates the effects of immunosuppression on COVID-19 outcomes in patients with autoimmune diseases and solid organ transplants. Using a comprehensive nationwide database of confirmed COVID-19 cases, we conducted a detailed analysis of mortality rates, ICU admissions, and the need for respiratory support. By examining the roles of immunosuppressive therapies and vaccination, this study aims to provide insights that can inform treatment protocols and vaccination strategies for immunocompromised patients during the COVID-19 pandemic.

## 2. Methods

### 2.1. Patient Population and Cohorts

This study used a matched cohort design and data from the K-COV-N cohort (Korea Disease Control and Prevention Agency-COVID19-National Health Insurance Service cohort), which includes the mandatory reporting of all patients with COVID-19 in Korea. This ensured that there were no omissions of diagnosed individuals from medical institutions in the dataset. The study duration was from 8 October 2020, to 31 December 2021. The inclusion criteria for the study were individuals who were 18 years of age or older and had a positive COVID-19 PCR or rapid antigen test. The exclusion criteria were individuals under 18 years of age, patients with cancer diagnosed within the last year, patients with immune deficiency, and individuals who were taking steroid prednisolone equivalent doses of 20 mg/day or higher but were not autoimmune disease or transplantation patients. At least one patient’s observation period had to be at least 3 months to ensure an adequate window period for analysis.

### 2.2. Definitions

The individual index date was defined as the date of the COVID-19 diagnosis for each patient. Current medication use was defined as any medication taken within 3 months before the index date. Patients who had not taken corticosteroids or oral immunosuppressants for three months before the index date and had no prescriptions for immunosuppressants during the same period were considered to have no immunosuppressant drug use. Patients with cancer were defined as those who had been diagnosed with cancer within the last year. Autoimmune disease patients were defined as those who had ICD codes twice or more within the last year and were taking at least one immunosuppressant drug (Detailed code lists were included in Supplementary Table S1). Transplantation patients were defined as those who had ICD codes for transplantation at least once in their lifetime and who were taking at least one immunosuppressant drug (detailed code lists are included in Supplementary Table S2). Comorbidities included diabetes mellitus (ICD codes E10–E14), hypertension (ICD codes I10–I15), cardiovascular disease (ICD codes I20–I25 and I50), stroke (ICD codes I60–I69), and chronic kidney disease (ICD code N18). High-dose steroid use was defined as a daily dose equivalent to 20 mg or higher of prednisolone, and low-dose steroid use was defined as a daily dose of prednisolone equivalent to less than 20 mg/day.

### 2.3. Statistical Analysis

The demographic and clinical characteristics of the cohorts were summarized using the mean and standard deviation for continuous variables, and frequencies and percentages for categorical variables. To ensure a precise comparison of COVID-19 outcomes between autoimmune patients and transplant recipients, two separate matched control cohorts were created. This distinction reflects the inherent differences in baseline characteristics and treatment protocols between these two patient groups. Cohort A comprised patients with autoimmune diseases, while Cohort B included transplant recipients. Although the inclusion and exclusion criteria for selecting controls in both cohorts were similar—excluding individuals with autoimmune diseases, transplant histories, immune deficiencies, cancer, or recent use of immunosuppressants—propensity score matching (PSM) introduced differences tailored to each group. For each cohort, PSM was employed to select control individuals matched to the autoimmune or transplant patients based on age, sex, comorbidities, Charlson Comorbidity Index (CCI), region, and socioeconomic status. This approach ensured that the control populations were closely comparable to their respective patient groups while remaining free from conditions that could confound COVID-19 outcomes. The separation of Cohort A and B controls allowed for a more accurate evaluation of outcomes specific to each patient group, recognizing that differences in demographics and comorbidity burden could otherwise skew results if analyzed together. We used a greedy nearest-neighbor algorithm with a caliper width of 0.001 on the propensity scores derived from these covariates to identify the controls. The process achieved a standardized mean difference of less than 10% for all matched variables in cohorts A and B, demonstrating the effectiveness of the matching procedure.

The primary outcome of this study was in-hospital mortality rate. Secondary outcomes included 28-day mortality, ICU admission, administration of oxygen, high-flow nasal cannula oxygen therapy (HFNC), mechanical ventilation (MV), and extracorporeal membrane oxygenation (ECMO). The adjusted odds ratios (aORs) with 95% confidence intervals (CIs) for each outcome were calculated for both cohorts. The minimally adjusted OR was calculated with adjusted covariates including only age and sex. The fully adjusted OR was calculated by adjusting for all covariates.

Subgroup analysis was conducted to assess the effects of immunocompromise severity, defined by steroid usage (categorized as no steroid, low-dose steroid, or high-dose steroid) and the number of immunosuppressant medications (1, 2, or 3 or more medications), and vaccination history, categorized by the number of vaccine doses received (unvaccinated, 1 dose, 2 doses, or 3 or more doses), on COVID-19 outcomes. These clinical factors, predetermined for stratification, facilitated the investigation of outcome disparities. Multivariable logistic regression was utilized to compute adjusted odds ratios (ORs) with 95% confidence intervals (CIs) for each subgroup, controlling for confounders including age, sex, comorbidities, and socioeconomic status.

Statistical analysis utilized SAS (version 9.4) and R (version 4.2). A *p*-value of 0.05 was set as the threshold for statistical significance. This report followed the Strengthening the Reporting of Observational Studies in Epidemiology (STROBE) guidelines.

## 3. Results

### 3.1. Patient Characteristics

This study enrolled 323,890 patients, including 167,732 males (51.79%) and 156,158 females (48.21%). The distribution of the groups was as follows: 188,136 patients in the control group, 135,341 patients with autoimmune diseases, and 413 transplant recipients. The age distribution showed the highest proportion of transplant patients in the 60–69 years age bracket (34.62%), whereas the control and autoimmune cohorts had a majority in the 18–29 years age range (23.65% and 19.98%, respectively). The transplantation cohort exhibited a higher comorbidity burden, with 19.37% having a CCI ≥ 4. No significant variances were observed in the regional distribution or income levels across cohorts. Steroid use was prevalent in the autoimmune cohort (94,876 patients, 70.1%), with 60% of patients receiving high doses. Among the transplant recipients, 258 (62.5%) were steroid users, with a notable percentage concurrently taking multiple immunosuppressants: 79.42% on tacrolimus and 74.58% on mycophenolate mofetil (MMF). Most transplantations were renal (247 patients, 71.8%), followed by liver (57 patients, 16.57%), lung (22 patients, 6.4%), and heart (17 patients, 4.94%). Combined heart-lung transplants were the least common, with one case (0.29%). Vaccination rates showed that more than 40% of the individuals in all cohorts received at least three doses, with the highest proportion of unvaccinated individuals in the transplantation cohort (91 patients, 22.03%). In contrast, the control and autoimmune groups had approximately 11–12% unvaccinated individuals (Table 1).

After propensity score matching, matched cohort A (*n* = 120,766) was compared to the autoimmune disease cohort and matched cohort B (*n* = 688) to the transplantation cohort. The baseline covariates demonstrated parity between the cohorts according to the standardized mean difference (Table 2, Supplementary Figure S1).

### 3.2. Outcomes

In the autoimmune disease cohort, the analysis indicated an increased risk of multiple complications. The aOR for in-hospital mortality was 2.749 (95% CI: 2.403–3.144, *p* < 0.0001), 28-day mortality 2.293 (95% CI: 1.889–2.785, *p* < 0.0001), and ICU admission 8.827 (95% CI: 7.689–10.134, *p* < 0.0001). Oxygen therapy (aOR: 5.119, 95% CI: 4.861–5.390, *p* < 0.0001), HFNC (aOR: 27.220, 95% CI: 21.393–34.634, *p* < 0.0001), MV, and ECMO requirements were also elevated. The transplantation cohort exhibited increased aORs for in-hospital mortality (7.567, 95% CI: 2.867–19.974, *p* < 0.0001) and ICU admission (17.109, 95% CI: 8.500–34.435, *p* < 0.0001), with a nonsignificant trend towards increased 28-day mortality (4.866, 95% CI: 0.903–26.230, *p* = 0.0654). Increased requirements for oxygen therapy, HFNC, MV, and ECMO were also observed (Table 3).

### 3.3. Subgroup Analysis

Steroid dosage was a determining factor for clinical outcomes in both the autoimmune disease and transplantation cohorts. In patients with autoimmune diseases, high-dose steroids increased the risk of in-hospital mortality (aOR 1.332, 95% CI: 1.192–1.488, *p* < 0.001), 28-day mortality (aOR 3.859, 95% CI: 3.232–4.609, *p* < 0.001), ICU admission (aOR 17.104, 95% CI: 14.192–20.613, *p* < 0.001), oxygen requirement (aOR 2.283, 95% CI: 1.733–3.008, *p* < 0.001), HFNC (aOR 4.348, 95% CI: 4.146–4.561), and MV (aOR 6.401, 95% CI: 5.267–7.78, *p* < 0.001). Low-dose steroid use was associated with marginally better outcomes, except for in-hospital mortality (aOR 0.911, 95% CI: 0.844–0.984, *p* = 0.017). Transplant recipients receiving high-dose steroids showed a higher, but not statistically significant, OR for in-hospital mortality (aOR 2.053, 95% CI: 0.896–4.705, *p* = 0.089). However, the risk for ICU admission was significantly increased (aOR 8.696, 95% CI: 3.21–23.561, *p* < 0.001), as was the risk for 28-day mortality (aOR 3.864, 95% CI: 1.33–11.23, *p* = 0.013) and the need for MV (aOR 3.005, 95% CI: 1.217–7.421, *p* = 0.017). Low-dose steroid use in transplant recipients was associated with outcomes similar to those of the control group.

The number of immunosuppressant medications was a significant determinant of the outcomes. For patients with autoimmune diseases taking three or more medications, the aOR for in-hospital mortality was not significant (1.825, 95% CI: 0.224–14.899, *p* = 0.574). However, the aOR for 28-day mortality (44.758, 95% CI: 12.354–162.152, *p* < 0.001) and ICU admission (55.022, 95% CI: 17.734–170.716, *p* < 0.001) was significant. For HFNC and MV, aORs were 3.829 (95% CI: 2.485–5.899) and 224 (95% CI: 91.253–549.853), respectively. In the transplantation cohort, the ICU admission risk increased significantly (aOR 55.022, 95% CI: 17.734–170.716, *p* < 0.001), as did the need for HFNC (aOR 3.463, 95% CI: 1.633–7.346, *p* = 0.001) and MV (aOR 46.134, 95% CI: 13.91–153.01, *p* < 0.001).

**Figure 1:**
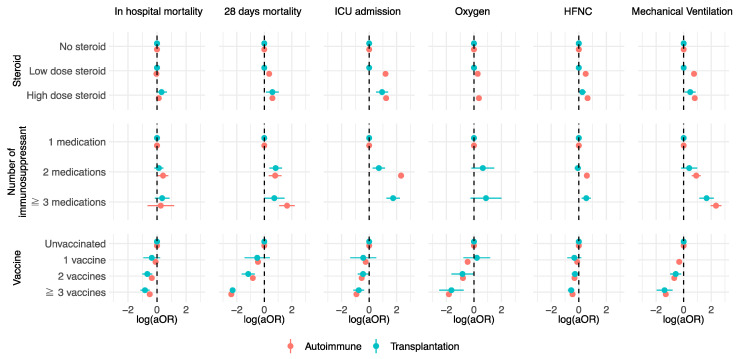
The subgroup analysis of each outcome with steroid doses, number of immunosuppressant, and vaccination rate.

The vaccination status significantly influenced patient outcomes. In the autoimmune cohort, the aOR for in-hospital mortality decreased with subsequent vaccine doses as follows: one dose (aOR 0.793, 95% CI: 0.651–0.966, *p* = 0.021), two doses (aOR 0.44, 95% CI: 0.396–0.489, *p* < 0.001), and three or more doses (aOR 0.316, 95% CI: 0.287–0.348, *p* < 0.001). The trend was similar for 28-day mortality, decreasing to an aOR of 0.347 (95% CI: 0.259–0.463, *p* < 0.001), 0.147 (95% CI: 0.127–0.171, *p* < 0.001), and 0.004 (95% CI: 0.003–0.006, *p* < 0.001) with one, two, and three or more doses, respectively. In transplant recipients, the in-hospital mortality aORs were 0.438 (95% CI: 0.116–1.652, *p* = 0.223), 0.217 (95% CI: 0.098–0.481, *p* < 0.001), and 0.149 (95% CI: 0.073–0.306, *p* < 0.001) for one, two, and three or more doses, respectively. For 28-day mortality, aORs were 0.308 (95% CI: 0.037–2.545, *p* = 0.274), 0.068 (95% CI: 0.023–0.203, *p* < 0.001), and 0.005 (95% CI: <0.001–0.028, *p* < 0.001) for one, two, and three or more doses, respectively. The autoimmune disease cohort showed effectiveness after the first dose, whereas the transplantation cohort showed effectiveness after the second dose. Figure 1 depicts the log-transformed aORs for outcomes per subgroup.

## 4. Discussion

In this study, we observed that patients with immunocompromised patients, encompassing those with autoimmune diseases and solid organ transplant recipients, exhibited heightened risks of severe COVID-19 and increased mortality rates. Subgroup analysis suggested a dose-response relationship where higher steroid dosages and more extensive immunosuppressant use were associated with poorer outcomes. Notably, vaccination emerged as a protective factor; for patients with autoimmune diseases, even a single vaccine dose was linked to better outcomes, whereas for transplant patients, a minimum of two doses were necessary to observe a similar benefit.

Our study demonstrated higher estimates than previous studies in the field (autoimmune disease, OR 2.29 (95% CI 1.89–2.79); transplantation, OR 4.87 (95% CI 0.90–26.23)). In addition, other outcomes, such as oxygen, HFNC, mechanical ventilation, ICU admission, and ECMO, were significantly poorer than in healthy controls. A previous study [10] focusing on autoimmune inflammatory rheumatic diseases reported an OR of 1.26–1.71 for severe COVID-19 and 1.69–1.87 for COVID-19-related deaths. The study examining 2307 solid organ transplantation recipients reported that transplantation increased only the risk of hospital admission (RR 1.22, 95% CI: 1.1–1.34) and acute kidney injury (RR 1.73, 95% CI: 1.53–1.96). However, the risk ratio for 30-day mortality, 60-day mortality, mechanical ventilator use, and ICU admission did not notably increase [13]. In contrast, in another study, the hazard ratio (HR) for COVID-19 mortality was 3.53 (95% CI 2.77–4.49) for organ transplant recipients, 1.19 (95% CI 1.11–1.27) for conditions such as rheumatoid arthritis, lupus, and psoriasis, and 2.21 (95% CI 1.68–2.90) for other immunosuppressive conditions [8]. This difference stems from the fact that we conducted a matched-pair analysis focusing on patients with COVID-19 who did not present with cancer, autoimmune diseases, or other immunocompromised conditions. Unlike a previous study that focused on a Korean population but included individuals with cancer or a history of steroid use, our study strictly excluded such populations, potentially leading to the observed elevated OR [7].

In the present study, patients who underwent transplantation exhibited a higher OR for adverse outcomes compared with those with autoimmune conditions. Although direct comparisons are challenging due to differences in baseline cohort covariates, it is evident that the transplantation cohort faced a greater risk of poor outcomes. This increased risk appears to stem from several factors, including the more extensive use of immunosuppressants, the higher prevalence of comorbidities, and the age distribution of the transplant group, which had a higher proportion of patients in their 60s and 70s compared to other cohorts. While age is an important factor, we believe the increased risk in transplant recipients is primarily driven by the degree of immunosuppression and comorbidities, rather than age alone. Our study found a similar level of risk in an autoimmune disease cohort treated with high-dose steroids, with an OR of approximately 3.8. This finding is consistent with previous research, which reported that patients with autoimmune inflammatory rheumatic diseases on high-dose steroids had an increased risk of severe COVID-19 (OR 1.76, 95% CI 1.06–2.96) and COVID-19-related death (OR 3.34, 95% CI 1.23–8.90). In contrast, the use of disease-modifying antirheumatic drugs did not show a significant difference in the risk of severe COVID-19 or COVID-19-related deaths [10]. In our study, the number of immunosuppressant medications was found to impact outcomes in a dose-dependent manner. This aligns with findings from a study investigating the impact of immunosuppressants on COVID-19 outcomes. Except for Azathioprine, other immunosuppressants, such as mTOR inhibitors, mycophenolate and its derivatives, steroids, and calcineurin inhibitors, were associated with increased odds ratios (ORs) of 5.38, 2.13, and 1.60, respectively [31].

This study suggested that vaccination improves outcomes in immunocompromised patients in a dose-dependent manner. It aligns with previous studies showing that vaccinated immunocompromised patients experience a reduced incidence of COVID-19 [32] and a lower risk of COVID-19-related hospitalization [6,33]. Our study indicates that even a single vaccine dose can confer a certain degree of immunization against severe outcomes in these patients. Specifically, individuals with autoimmune diseases showed a reduction in severe COVID-19 cases after receiving just one vaccine dose. In contrast, transplant recipients only demonstrated a significant protective effect against COVID-19 after receiving two or more doses. This aligns with findings that immunosuppressants impair the effectiveness of vaccination [34,35,36,37], highlighting the need for booster doses [24]. However, subsequent vaccinations have shown to reduce mortality and the risk of severe disease progression, potentially strengthening the rationale for continued vaccination in transplant patients. This is particularly important given the higher degree of immunosuppression in transplant recipients compared to patients with autoimmune diseases, which may contribute to the differential impact of the initial vaccine dose.

Understanding the national vaccination landscape during the study period is crucial for interpreting our findings. By the end of 2021, 86.1% of the population had received at least one dose, 83.2% had completed two doses, and 34.4% had received a booster. AstraZeneca was the first vaccine introduced in February 2021, accounting for 25.23% of first doses. Pfizer followed, becoming the most widely used vaccine, contributing to 56.03% of first doses and 54.82% of second doses. Moderna, introduced in the second quarter, accounted for 15.33% of first doses, while Janssen, a single-dose vaccine, was administered to 3.41% of the population. Despite vaccines being developed for earlier variants like Alpha and Beta, they continued to offer strong protection against severe disease and hospitalization [38,39].

During the study period, the dominant SARS-CoV-2 variants in South Korea were Alpha, Beta, Gamma, and Delta [40]. Delta, which became prevalent from mid-2021, was associated with significantly higher transmissibility and severity, including a 120% increased risk of hospitalization, a 287% increased risk of ICU admission, and a 137% increased risk of death compared to earlier variants [41]. Although Omicron emerged in December 2021, it remained a minor variant during this period. Regardless of the circulating variants, immunocompromised patients remained highly vulnerable to severe outcomes, underscoring the importance of vaccination in this population.

The strength of this study lies in its nationwide design. Moreover, its strict inclusion criteria, which exclude cancer and other immunocompromised statuses besides autoimmune disease and transplantation conditions, feature a large sample size drawn from a nationwide cohort, rigorous application of PSM, and adjustments for confounding factors such as comorbidities and geographical location where patients resided. In addition, our comprehensive analysis allowed us to examine various outcomes, including the use of oxygen, HFNC, mechanical ventilation, and ECMO.

Our study had several notable limitations. The retrospective nature of our data inherently introduced potential biases and challenges in establishing causality. We attempted to counter these concerns by employing PSM in our analysis. A significant constraint was our inability to specify the types of vaccines used and the timing of the vaccination. Considering that different vaccines may elicit varying immune responses, it is difficult to attribute variations in outcomes to specific vaccine types. Additionally, our dataset did not include detailed information on specific COVID-19 treatments. Based on South Korean guidelines (1 December 2021), Remdesivir and Regdanvimab were recommended for severe and high-risk cases, respectively, with limited use of monoclonal antibodies and immune modulators [42,43]. This limited treatment availability may have influenced the outcomes in our cohort. Moreover, while our claims data allowed us to verify treatments, such as oxygen, HFNC, and MV, they did not provide insights into their intensity or duration. To gauge the severity of COVID-19, we relied on the application of various modalities such as oxygen, HFNC, MV, and ECMO. However, the exact level or frequency of oxygen or MV that greatly influences patient outcomes remains elusive. Additionally, our study predominantly focused on overall mortality, neglecting a specific emphasis on COVID-19 related deaths. Consequently, we observed associations between patient groups and mortality rates; however, our data did not conclusively attribute these deaths to COVID-19.

## 5. Conclusions

Immunocompromised patients are at a significantly higher risk of severe COVID-19 outcomes, highlighting a key healthcare inequality compared to the general population. This risk is high by the use of steroids and immunosuppressants but can be mitigated by reducing their use and prioritizing vaccination. Our study demonstrates that immunization plays a crucial role in improving prognosis: one or more doses significantly reduced severe outcomes in autoimmune patients, while transplant recipients required at least two doses for similar protection. Tailored vaccination strategies based on individual immunosuppression levels are essential to reducing these disparities and improving outcomes.

## Figures and Tables

**Table 1:** Baseline characteristics of patients across total population and individual cohorts.

		Control (N = 188,136)	Autoimmune (N = 135,341)	Transplantation (N = 413)
Sex				
	Male	106,595 (56.66)	60,874 (44.98)	263 (63.68)
	Female	81,541 (43.34)	74,467 (55.02)	150 (36.32)
Age group (years), N (%)				
	18–29	44,494 (23.65)	27,042 (19.98)	27 (6.54)
	30–39	36,990 (19.66)	23,183 (17.13)	24 (5.81)
	40–49	36,794 (19.56)	21,907 (16.19)	52 (12.59)
	50–59	32,280 (17.16)	22,383 (16.54)	127 (30.75)
	60–69	25,896 (13.76)	24,686 (18.24)	143 (34.62)
	70–79	7800 (4.15)	10,503 (7.76)	39 (9.44)
	80–89	3012 (1.6)	4633 (3.42)	1 (0.24)
	≥90	870 (0.46)	1004 (0.74)	0 (0)
Comorbidity, N (%)				
	Diabetes	31,540 (16.76)	39,409 (29.12)	274 (66.34)
	Cardiovascular disease	13,820 (7.35)	19,998 (14.78)	167 (40.44)
	Cerebrovascular disease	5045 (2.68)	7011 (5.18)	34 (8.23)
	Hypertension	38,774 (20.61)	42,428 (31.35)	274 (66.34)
	Chronic Kidney disease	1378 (0.73)	2587 (1.91)	271 (65.62)
CCI, N (%)				
	0–1	182,179 (96.83)	125,431 (92.68)	188 (45.52)
	2	3862 (2.05)	6071 (4.49)	88 (21.31)
	3	1258 (0.67)	2329 (1.72)	57 (13.8)
	≥4	837 (0.44)	1510 (1.12)	80 (19.37)
Region				
	Seoul	75,427 (40.09)	50,305 (37.17)	159 (38.5)
	Gyeonggi-do	55,247 (29.37)	39,383 (29.1)	124 (30.02)
	Incheon	11,433 (6.08)	8005 (5.91)	25 (6.05)
	Busan	5915 (3.14)	6769 (5)	15 (3.63)
	Gyeongsangnam-do	5457 (2.9)	4880 (3.61)	9 (2.18)
	Chungcheongnam-do	4790 (2.55)	3482 (2.57)	6 (1.45)
	Daegu	4596 (2.44)	3616 (2.67)	11 (2.66)
	Gyeongsangbuk-do	3690 (1.96)	2874 (2.12)	11 (2.66)
	Gangwon-do	3658 (1.94)	2551 (1.88)	7 (1.69)
	Daejeon	3287 (1.75)	3026 (2.24)	8 (1.94)
	Chungcheongbuk-do	2965 (1.58)	2252 (1.66)	7 (1.69)
	Jeollabuk-do	2896 (1.54)	1905 (1.41)	6 (1.45)
	Gwangju	2366 (1.26)	1397 (1.03)	6 (1.45)
	Ulsan	1720 (0.91)	1667 (1.23)	5 (1.21)
	Jeollanam-do	1703 (0.91)	1323 (0.98)	7 (1.69)
	Jeju-do	1340 (0.71)	1004 (0.74)	3 (0.73)
	Sejong	583 (0.31)	389 (0.29)	1 (0.24)
Household income (KRW)				
	Low (0–39)	57,410 (30.52)	43,131 (31.87)	135 (32.69)
	Middle (40–79)	76,446 (40.63)	49,705 (36.73)	147 (35.59)
	High (80–100)	50,976 (27.1)	40,338 (29.8)	122 (29.54)
Steroids				
	No steroid	188,136 (100%)	40,465 (29.9%)	155 (37.5%)
	Low dose steroid	0 (0%)	13,705 (10.1%)	59 (14.3%)
	High dose steroid	0 (0%)	81,171 (60%)	199 (48.2%)
Immunosuppressant drugs				
	Cyclosporin	-	820 (0.61)	52 (12.59)
	Tacrolimus	-	353 (0.26)	328 (79.42)
	MMF	-	234 (0.17)	308 (74.58)
	Azathioprine	-	375 (0.28)	6 (1.45)
	Sirolimus	-	6 (0)	17 (4.12)
	Simulect	-	18 (0.01)	46 (11.14)
	Alemtuzumab	-	0 (0)	0 (0)
	Rituximab	-	24 (0.02)	19 (4.6)
	Everolimus	-	5 (0)	11 (2.66)
Transplantation				
	Kidney	-	-	311 (75.3)
	Liver	-	-	60 (14.53)
	Lung	-	-	24 (5.81)
	Heart	-	-	17 (4.12)
	Heart and lung	-	-	1 (0.24)
Vaccination (doses)				
	0	23,699 (12.6%)	15,430 (11.4%)	91 (22.03%)
	1	6972 (3.7%)	4832 (3.6%)	9 (2.18%)
	2	74,780 (39.7%)	50,005 (37.0%)	130 (31.48%)
	≥3	82,685 (43.9%)	65,074 (48.1%)	183 (44.31%)

**Table 2:** Characteristics of post-propensity score matching demographics in control A and autoimmune disease cohort; control B and transplantation cohort.

		Control_A (N = 120,766)	Autoimmune (N = 120,766)	Control_B (N = 688)	Transplantation (N = 344)
Sex					
	Male	57,648 (47.74)	57,423 (47.55)	421 (61.19)	212 (61.63)
	Female	63,118 (52.26)	63,343 (52.45)	267 (38.81)	132 (38.37)
Age group (years), N (%)					
	18–29	26,268 (21.75)	26,137 (21.64)	37 (5.38)	22 (6.4)
	30–39	21,953 (18.18)	21,956 (18.18)	38 (5.52)	20 (5.81)
	40–49	21,396 (17.72)	20,684 (17.13)	62 (9.01)	42 (12.21)
	50–59	20,990 (17.38)	20,308 (16.82)	152 (22.09)	106 (30.81)
	60–69	19,926 (16.5)	20,499 (16.97)	221 (32.12)	120 (34.88)
	70–79	6733 (5.58)	7673 (6.35)	102 (14.83)	33 (9.59)
	80–89	2708 (2.24)	2874 (2.38)	51 (7.41)	1 (0.29)
	≥90	792 (0.66)	635 (0.53)	25 (3.63)	0 (0)
Comorbidity, N (%)					
	Diabetes	28,875 (23.91)	28,742 (23.8)	473 (68.75)	213 (61.92)
	Cardiovascular disease	12,879 (10.66)	13,099 (10.85)	270 (39.24)	119 (34.59)
	Cerebrovascular disease	4504 (3.73)	4541 (3.76)	73 (10.61)	31 (9.01)
	Hypertension	32,781 (27.14)	32,687 (27.07)	497 (72.24)	213 (61.92)
	Chronic Kidney disease	1308 (1.08)	1340 (1.11)	409 (59.45)	204 (59.3)
CCI, N (%)					
	0–1	115,092 (95.3)	114,984 (95.21)	356 (51.74)	184 (53.49)
	2	3673 (3.04)	37,745 (31.25)	134 (19.48)	68 (19.77)
	3	1202 (1)	1174 (0.97)	72 (10.47)	38 (11.05)
	≥4	799 (0.66)	833 (0.69)	126 (18.31)	54 (15.7)
Region					
	Seoul	46,164 (38.23)	46,331 (38.36)	312 (45.35)	144 (41.86)
	Gyeonggi-do	35,531 (29.42)	35,555 (29.44)	193 (28.05)	98 (28.49)
	Incheon	7333 (6.07)	7194 (5.96)	37 (5.38)	18 (5.23)
	Busan	5082 (4.21)	5076 (4.2)	24 (3.49)	10 (2.91)
	Gyeongsangnam-do	4132 (3.42)	4132 (3.42)	13 (1.89)	9 (2.62)
	Daegu	3178 (2.63)	3117 (2.58)	20 (2.91)	8 (2.33)
	Chungcheongnam-do	3131 (2.59)	3083 (2.55)	7 (1.02)	6 (1.74)
	Daejeon	2625 (2.17)	2535 (2.1)	7 (1.02)	6 (1.74)
	Gyeongsangbuk-do	2429 (2.01)	2492 (2.06)	12 (1.74)	9 (2.62)
	Gangwon-do	2128 (1.76)	2180 (1.81)	10 (1.45)	5 (1.45)
	Chungcheongbuk-do	1943 (1.61)	1953 (1.62)	13 (1.89)	6 (1.74)
	Jeollabuk-do	1687 (1.4)	1666 (1.38)	13 (1.89)	6 (1.74)
	Ulsan	1434 (1.19)	1385 (1.15)	6 (0.87)	3 (0.87)
	Gwangju	1223 (1.01)	1276 (1.06)	8 (1.16)	5 (1.45)
	Jeollanam-do	1081 (0.9)	1105 (0.91)	6 (0.87)	4 (1.16)
	Jeju-do	883 (0.73)	881 (0.73)	2 (0.29)	3 (0.87)
	Quarantine	414 (0.34)	457 (0.38)	2 (0.29)	3 (0.87)
	Sejong	368 (0.3)	348 (0.29)	3 (0.44)	1 (0.29)
Household income					
	Low (0–39)	38,480 (31.86)	38,642 (32)	249 (36.19)	117 (34.01)
	Middle (40–79)	45,825 (37.95)	46,260 (38.31)	228 (33.14)	121 (35.17)
	High (80–100)	36,461 (30.19)	35,864 (29.7)	211 (30.67)	106 (30.81)
Steroid group	No steroid	120,766 (100)	37,227 (30.83)	688 (100)	129 (37.5)
	High dose	0 (0)	71,576 (59.27)	0 (0)	166 (48.26)
	Low dose	0 (0)	11,963 (9.91)	0 (0)	49 (14.24)
Immunosuppressant drugs					
	Cyclosporin	0 (0)	735 (0.61)	0 (0)	48 (13.95)
	Tacrolimus	0 (0)	241 (0.2)	0 (0)	268 (77.91)
	Mycophenolate Mofetil	0 (0)	145 (0.12)	0 (0)	255 (74.13)
	Azathioprine	0 (0)	336 (0.28)	0 (0)	3 (0.87)
	Sirolimus	0 (0)	2 (0)	0 (0)	12 (3.49)
	Simulect	0 (0)	12 (0.01)	0 (0)	39 (11.34)
	Alemtuzumab	0 (0)	0 (0)	0 (0)	0 (0)
	Rituximab	0 (0)	17 (0.01)	0 (0)	13 (3.78)
	Everolimus	0 (0)	4 (0)	0 (0)	11 (3.2)
Transplantation					
	Kidney	-	-	0 (0)	247 (71.8)
	Heart	-	-	0 (0)	17 (4.94)
	Lung	-	-	0 (0)	22 (6.4)
	Heart and lung	-	-	0 (0)	1 (0.29)
	Liver	-	-	0 (0)	57 (16.57)
Autoimmune disease	Rheumatoid arthritis	-	7452 (6.17)	-	9 (2.62)
	Systemic connective tissue disorders	-	3077 (2.55)	-	12 (3.49)
	Raynaud’s syndrome	-	272 (0.23)	-	2 (0.58)
	Sarcoidosis	-	18 (0.01)	-	0 (0)
	Autoimmune hemolytic anemia	-	104 (0.09)	-	1 (0.29)
	Immune thrombocytopenic purpura	-	343 (0.28)	-	0 (0)
	Guillain-Barre syndrome	-	51 (0.04)	-	0 (0)
	Myasthenia gravis	-	121 (0.1)	-	0 (0)

**Table 3:** The odds ratio for the clinical outcomes in both the autoimmune disease cohort and the transplantation cohort, compared to their respective matched control groups.

Outcomes		Autoimmune	*p*-Value	Transplantation	*p*-Value
In hospital mortality	Minimal adjusted OR	2.618 (2.293–2.988)	<0.0001	5.336 (2.268–12.552)	0.0001
	Fully adjusted OR	2.749 (2.403–3.144)	<0.0001	7.567 (2.867–19.974)	<0.0001
28 days mortality	Minimal adjusted OR	2.225 (1.835–2.698)	<0.0001	4.553 (1.086–19.096)	0.0382
	Fully adjusted OR	2.293 (1.889–2.785)	<0.0001	4.866 (0.903–26.230)	0.0654
ICU admission	Minimal adjusted OR	8.679 (7.561–9.962)	<0.0001	15.033 (7.752–29.152)	<0.0001
	Fully adjusted OR	8.827 (7.689–10.134)	<0.0001	17.109 (8.500–34.435)	<0.0001
Oxygen	Minimal adjusted OR	5.011 (4.761–5.276)	<0.0001	8.515 (5.573–13.011)	<0.0001
	Fully adjusted OR	5.119 (4.861–5.390)	<0.0001	10.670 (6.730–16.917)	<0.0001
High flow nasal cannula (HFNC)	Minimal adjusted OR	26.555(20.873–33.783)	<0.0001	32.208 (12.365–83.896)	<0.0001
	Fully adjusted OR	27.220(21.393–34.634)	<0.0001	35.452 (13.346–94.174)	<0.0001
Mechanical Ventilation (MV)	Minimal adjusted OR	19.543 (14.042–27.200)	<0.0001	32.862 (12.188–88.602)	<0.0001
	Fully adjusted OR	20.044 (14.399–27.903)	<0.0001	16.200 (16.078–16.322)	<0.0001
ECMO	Minimal adjusted OR	-	-	75.492 (18.71–304.6)	<0.0001
	Fully adjusted OR	8.569 (8.442–8.698)	<0.0001	76.327 (18.915–307.994)	<0.0001

## Data Availability

Deidentified data licensed for this analysis were obtained from the Korean National Health Insurance Service (NHIS). Due to privacy regulations, these data are not publicly available. However, they may be made available upon reasonable request, subject to approval from the NHIS. Researchers interested in accessing the data can apply through the NHIS bigdata platform system (nhiss.nhis.or.kr).

## Supplementary Materials

The following supporting information can be downloaded at: https://www.mdpi.com/article/10.3390/vaccines12101190/s1, Figure S1: Love plot for each group; Table S1: List of ICD-10 Codes for Autoimmune Diseases; Table S2: List of ICD-10 Codes for Transplantation.
