# Supplementary material for: Impact of Immunosuppressants and Vaccination on COVID-19 Outcomes in Autoimmune Patients and Solid Organ Transplant Recipients: A Nationwide Propensity Score-Matched Study
Source: Vaccines (Basel). 2024 Oct 18;12(10):1190. doi: 10.3390/vaccines12101190 (PMC11512354; doi:10.3390/vaccines12101190)

Supplementary material

Table 1. List of ICD-10 Codes for Autoimmune Diseases

**Disease**	**Codes**
Systemic autoimmune disease	
Rheumatoid arthritis	M06.9
Systemic connective tissue disorders	M30-M36
Raynaud's syndrome	I73.0
Sarcoidosis	D86.9
Autoimmune hemolytic anemia	D59.0
Autoimmune hemolytic anemia	D59.1
Immune thrombocytopenic purpura	D69.2
Guillain-Barre syndrome	G61.0
Myasthenia gravis	G70.0
Autoimmune disease not elsewhere classified	D89
Organ specific autoimmune disease	
Hashimoto's thyroiditis	E06.3
Grave's disease	E05.0
Ankylosing spondylitis	M45.9
Celiac disease	K90.0
Inflammatory bowel disease	K50-K52
Psoriasis and psoriatic arthritis	L40
Autoimmune hepatitis	K75.4
Primary biliary cirrhosis	K74.3
Polyarteritis nodosa	M30.0
Pemphigus vulgaris	L10.0
Vasculitis limited to the skin unspecified	L95.9

Table 2. List of ICD-10 Codes for Transplantation

Organ	Codes
Kidney	Z94.0
Heart	Z94.1
Lung	Z94.2
Heart and lung	Z94.3
Liver	Z94.4

Figure 1. Love plot for each group

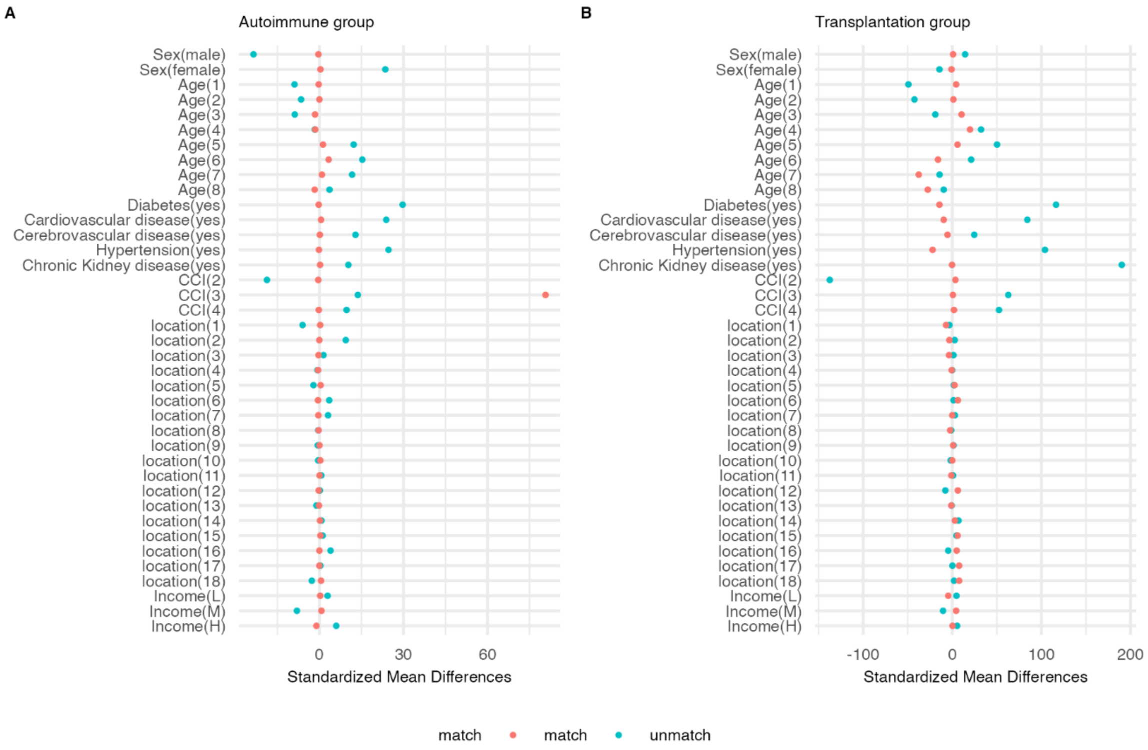

Supplement: Supplementary file 1 [file vaccines-12-01190-s001.zip › vaccines-3230157-supplementary.pdf]
